# Stem Cell-Derived Exosome in Cardiovascular Diseases: Macro Roles of Micro Particles

**DOI:** 10.3389/fphar.2018.00547

**Published:** 2018-05-31

**Authors:** Ye Yuan, Weijie Du, Jiaqi Liu, Wenya Ma, Lai Zhang, Zhimin Du, Benzhi Cai

**Affiliations:** ^1^Department of Clinical Pharmacy, The Second Affiliated Hospital of Harbin Medical University, Harbin, China; ^2^Department of Pharmacology, College of Pharmacy, Harbin Medical University, Harbin, China; ^3^Department of Pharmacology, College of Pharmacy, Mudanjiang Medical University, Mudanjiang, China

**Keywords:** exosomes, cardiovascular diseases (CVDs), stem cells, cell therapy, drug delivery, biomarkers

## Abstract

The stem cell-based therapy has emerged as the promising therapeutic strategies for cardiovascular diseases (CVDs). Recently, increasing evidence suggest stem cell-derived active exosomes are important communicators among cells in the heart via delivering specific substances to the adjacent/distant target cells. These exosomes and their contents such as certain proteins, miRNAs and lncRNAs exhibit huge beneficial effects on preventing heart damage and promoting cardiac repair. More importantly, stem cell-derived exosomes are more effective and safer than stem cell transplantation. Therefore, administration of stem cell-derived exosomes will expectantly be an alternative stem cell-based therapy for the treatment of CVDs. Furthermore, modification of stem cell-derived exosomes or artificial synthesis of exosomes will be the new therapeutic tools for CVDs in the future. In addition, stem cell-derived exosomes also have been implicated in the diagnosis and prognosis of CVDs. In this review, we summarize the current advances of stem cell-derived exosome-based treatment and prognosis for CVDs, including their potential benefits, underlying mechanisms and limitations, which will provide novel insights of exosomes as a new tool in clinical therapeutic translation in the future.

## Introduction

Cardiovascular diseases (CVDs) are still the leading causes of morbidity and mortality throughout the world. Although a proportion of CVDs is largely preventable, the incidence is increasing particularly in the low- and middle-income countries (Mendis et al., 2011; Roth et al., 2015). A gradual death/loss of cardiomyocyte has been characteristically observed in tissue remodeling especially across ischemic heart diseases (IHDs) (Olivetti et al., 1996). Over the past decades, investigators have recognized the importance of the regenerative medicine for treating and preventing cardiac diseases (Stayton et al., 2005). Transplantation of stem/progenitor cells has been well identified as one promising therapeutic strategy for CVDs demonstrated to replace the lost cardiomyocytes and improve contractility (Srivastava and Ivey, 2006; Li and Hacker, 2017). These cells include embryonic stem cells (ESCs) (Menasché et al., 2015), induced pluripotent stem cells (iPSCs) (Savla et al., 2014), mesenchymal stem cells (MSCs) (Golpanian et al., 2016; Majka et al., 2017), and cardiac stem/progenitor cells (Koudstaal et al., 2013; Wang W. E. et al., 2013). These new discoveries on the regenerative potential of stem/progenitor cells have led to an explosion in clinical investigation (Segers and Lee, 2008; Tang et al., 2013).

Although the improvement of stem cell transplantation has indeed exhibited beneficial effects for cardiac functions, it is widely agreed that the mechanisms of this protective action remain unclear due to low efficiency of trans-differentiation into cardiomyocytes after cell engraftment (Garbern and Lee, 2013). This stimulates us with great interest in exploring the novel and putative mechanisms of heart repair by stem/progenitor cells (Glembotski, 2017). Stem cells secreted molecules signal patient‘s cells/tissue to change their behavior. In addition to paracrine molecules, a range of secreted membrane-enclosed vesicles especially exosomes were of increasing interest, which are involved in multiple physiological processes (Conlan et al., 2017; Poe and Knowlton, 2017). Several studies have demonstrated beneficial effects of stem cell-derived exosomes on cardioprotection as a result of exosomes carrying and transferring a variety of contents to the injured cells (Kishore and Khan, 2016; Jung et al., 2017). We summarize in this review current mechanistic insights including their potential benefits, underlying mechanisms and limitations of stem cell-derived exosomes for healing the damaged heart and provide translational perspectives for the clinical treatment of patients with CVDs in the future.

## Biology and function of exosomes

The extracellular vesicles (EVs) released by almost all cell types are categorized into six main classes including exosomes, microvesicles, ectosomes, membrane particles, exosome-like vesicles and apoptotic bodies based on their multiple features such as sizes, shapes, origin and main protein markers (Table 1; Thery et al., 2001, 2006, 2009; Cobelli et al., 2017). Using centrifugation-based protocols, it is simple and fast to distinguish subpopulations of EVs (Crescitelli et al., 2013). This review mainly focuses on exosomes, nano-sized vesicles in the range of diameter from 30 to 100 nm, firstly discovered in the maturing mammalian reticulocyte (Thery et al., 2002).

**Table 1:** Characteristics of different types of secreted vesicle.

**Vesicle types**	**Size**	**Shape**	**Origin**	**The main markers**	**Lipids component**
Exosomes	50-100 nm	Cup shaped	Internal compartments (endosomes)	Tetraspanins (CD63/CD9); Alix; TSG101; MHCI; HSP70	Cholesterol, sphingomyelin, ceramide, lipid rafts, phosphatidylserine
Microvesicles	100–1,000 nm	Irregular	Plasma membrane	CD40; selectins (CD62); integrins	Phosphatidylserine
Ectosomes	50–200 nm	Spherical	Plasma membrane	CR1 and proteolytic enzymes; no CD63	Enriched in cholesterol and diacylglycerol; expose phosphatidylserine
Membrane particles	50–80 nm	Round	Plasma membrane	CD133; integrins; GRP94; no CD63	Unclear
Exosome-like vesicles	20–50 nm	Irregular	Internal compartments	TNFRI	No lipid rafts
Apoptotic bodies	50–500 nm	Heterogeneous	Unclear	Histones	Unclear

**Figure 1:**
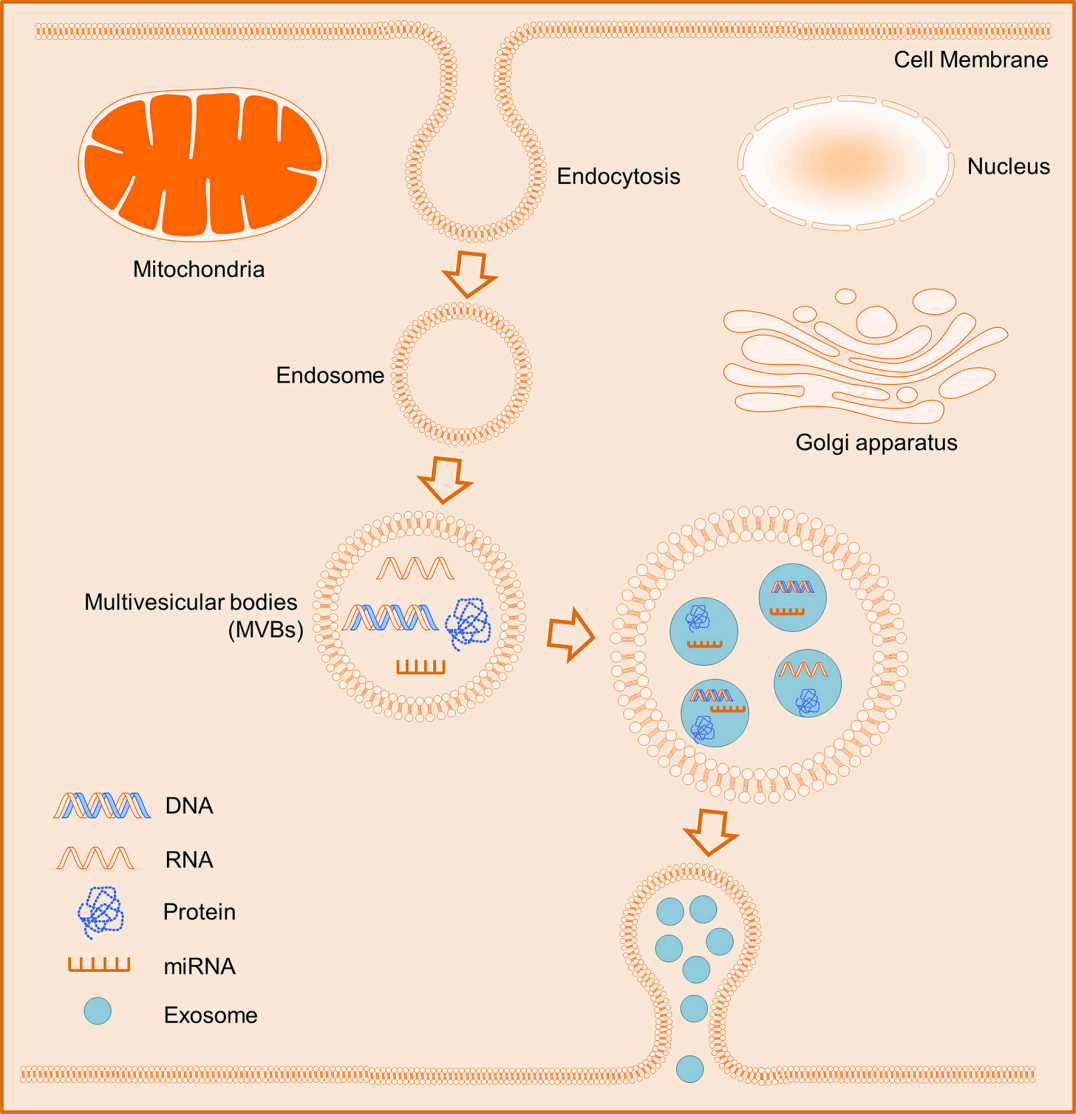
Schematic biogenesis of exosomes. The intracellular endosomes form by inward budding inside, and subsequently lead to the formation of multivesicular bodies (MVBs). The MVBs release eventually outside their internal exosomes containing some cell-specific DNAs, RNAs, miRNAs, or proteins.

In general, exosomes originate from intracellular endosomes by inward budding inside, which lead to the formation of multivesicular bodies (MVBs) and subsequently MVBs would fuse with the plasma membrane and then release outside its internal vesicles (Figure 1; Booth et al., 2006; Thery et al., 2009; Kowal et al., 2014). Integrins CD63 and CD81 are commonly recognized as specific surface markers for exosomes (Andreu and Yánez-Mó, 2014; Kowal et al., 2014). Whilst exosomes possess the capacity to deliver their contents to target cells comprising cell-specific proteins and nucleic acids including mRNA, ribosomal RNA (rRNA), microRNA (miRNA), long noncoding RNA (lncRNA), and variably some DNA (Valadi et al., 2007; Balaj et al., 2011). Thus, the release of exosomes is an important secreted fashion of contact between cells. Additionally, exosomes can interact with their target cells through multiple mechanisms i.e. (1) transmembrane proteins of exosomes interact directly with the signaling receptors of target cells; (2) exosomes fuse with the plasma membrane of recipient cells and deliver their contents into the cytosol; (3) exosomes internalize into the recipient cells. Interestingly, exosomes released into the circulation and bodily fluids are the major mediators of cell-cell communications, which are involved in the regulation of various biological processes of their target cells via exchange of genetic information and/or regulation of certain gene expressions (Camussi et al., 2010; Becker et al., 2016).

A growing body of evidence suggests exosomes have potential to become therapeutic and diagnostic guidelines for several diseases including liver diseases (Borrelli et al., 2018), kidney diseases (Lv et al., 2018), brain diseases (Rufino-Ramos et al., 2017) and CVDs (Bei et al., 2017). Notably, different contents have also displayed in healthy people and diseased patients (Jia et al., 2017; Li W. et al., 2017; Poe and Knowlton, 2017). Li and their colleagues have established a database including 58,330 circular RNAs (circRNAs), 15,501 lncRNAs and 18,333 mRNAs in human blood exosomes from 92 patients with different diseases (exoRBase http://www.exorbase.org/) (Li S. et al., 2017). It has been reported that tumor-secreted exosomes play an essential role in both primary tumor growth and metastatic evolution (Melo et al., 2014; Steinbichler et al., 2017). The blood in healthy subject was estimated to contain approximately 2,000 trillion exosomes but at least 4,000 trillion in patients with cancer (Steinbichler et al., 2017).

## Exosomes derived from cardiac cells

Exosomes were also interestingly identified to mediate communications among cardiac fibroblasts, endothelial cells and cardiomyocytes via delivering a wide variety of contents including proteins, nucleic acids (RNA and DNA) (Hirsch et al., 2013). This action is essential to support myocardium with oxygen and nutrients in normal heart, and in turn maintain heart homeostasis structural integrity. Upon pathological stimuli the secreted exosomes from cardiac cells will positively and negatively influence the function of neighbored cells (Chistiakov et al., 2016). Ronquist et al. provided new insights of communications between cardiomyocytes and fibroblasts. They transfected cardiomyocyte-derived exosomes containing 1,520 mRNA into fibroblasts, and of them, 333 genes expression level were changed in fibroblasts including 175 upregulated and 158 downregulated genes (Waldenstrom et al., 2012). Among these molecules in exosomes, miRNAs have recently attracted the most attention. For instance, Bang et al. showed that cardiac fibroblast exosomal-derived miR-21-3p was as a potent paracrine-acting RNA molecule that induces cardiomyocyte hypertrophy by inhibiting sorbin and SH3 domain containing 2 (SORBS2) and PDZ and LIM domain 5 (PDLIM5) expressions (Bang et al., 2014). Ribeiro-Rodrigues et al. determined that cardiomyocyte-derived exosomes upon ischemic condition promoted new vessel formation, and further study revealed this pro-angiogenic effect was partially as a result of relatively most expressed miR-143 and miR-222 in exosomes (Ribeiro-Rodrigues et al., 2017). Exosomes loaded with miR-146a are released by endothelial cells activated by the 16 kDa fragment of prolactin and target the erbB4 pathway in cardiac myocytes, resulting in myocyte loss and cardiomyopathy (Halkein et al., 2013); Other extracellular RNA (eRNA) species, including long non-coding RNA, may be released by inflammatory or endothelial cells upon activation of specific transcription factors (e.g., GATA2) or an ischaemic insult and modulate specific pathways in distant cells, such as pro-inflammatory TNF-alpha or p38-MAPK in cardiac myocytes (Hirsch et al., 2013). These cellular components in exosomes are not random but specific factors for their effects on cell-cell communications (Thery, 2011; Tkach and Thery, 2016). The cardiomyocyte-derived exosomes promote angiogenesis of endothelial cells (ECs) by stimulating pro-angiogenic factors in response to hypoxia (Louapre et al., 2005). Meanwhile, the ECs secreted exosome could enhance the angiogenic properties and increase the anti-apoptotic activity to cardiac progenitor cells (CPCs) to hypoxia stress by miRNA mediated signaling pathway (Huang and Zuo, 2014). The cardiac fibroblasts and cardiomyocyte are critically involved in heart healing and remodeling process after MI. The miRNAs-containing exosomes derived from these cells led them as the important regulatory players of development of cardiac hypertrophy and fibrosis (Bang et al., 2014). The exosome contained miRNAs have been shown to exhibit cardiac protective effects in infarcted myocardium by several mechanisms involving the regulation of intracardiac cells adaptive response (Castoldi et al., 2012). Cardiac telocyte cell, a novel type of stromal cells, stimulated the growth and differentiation of CSCs/CPCs during organogenesis, and improved cardiac function (Bei et al., 2016; Tao et al., 2016). Interestingly, transplantation of cardiac telocyte cells also showed enhanced angiogenesis and decreased cardiac fibrosis, which was involved in heart physiology and regeneration (Zhaofu and Dongqing, 2016). Cardiac telocyte cell-derived exosomes contain cardioprotective factors, which may exert beneficial effects on the damaged heart (Fertig et al., 2014; Marini et al., 2017).

## Exosome derived from stem cells for treating CVDs

Since the first successful bone marrow (BM) cell transplantation in 1968 (Bach et al., 1968), the regenerative medicine of cell therapy has been quickly emerged as alternative therapeutic strategies in many diseases, particularly in CVDs (Dimmeler et al., 2008; du Pré et al., 2013; Yu et al., 2017). The preclinical animal studies and some clinical trials have demonstrated that stem cell-based therapies could replace the lost cardiomyocytes and subsequently improve contractility and cure CVDs (Sanganalmath and Bolli, 2013). However, all these approaches have their own unique benefits and limitations. The low engraftment, immune rejection and tumorigenic potential limited the clinical application of stem cells therapy. Extensive studies have proven that in addition to direct trans-differentiation into cardiomyocytes, stem cells can repair injured tissue via paracrine mechanisms through exosomes (Gnecchi et al., 2008). Increasing evidence suggested that exosomes generated from stem cells exerted similarly protective and reparative properties with cellular counterparts of stem cell transplantation in repairing therapies (Baglio et al., 2012). Therefore, stem cells derived exosome-based therapy would be consider as a novel potential approach in the treatment of various cardiac diseases in the future (Singla, 2016; Suzuki et al., 2016; Prathipati et al., 2017; Rosca et al., 2017). In recent years, many researches have shifted the focus for treating CVDs from the transplanted multiple stem cells to their secreted exosomes.

### Exosomes from embryonic stem cells (ESCs) and ESC-derived cells

Embryonic stem cells (ESCs), derived from the undifferentiated inner mass cells of an embryo, can self-renew and differentiate into many types of cells, such as cardiomyocytes. Several studies have broadly observed ESC-derived cardiomyocytes could improve cardiac regeneration and enhance function in experimental animal models (Laflamme et al., 2007; Chong et al., 2014). Some factors, like Fucoidan promotes ESCs differentiation to cardiac lineages (Hamidi et al., 2014). Unfortunately, the ethical issues and the potential risk of tumor formation have limited the clinical application of ESCs. Recent studies have provided evidence about the importance of paracrine effects mediated intercellular communication within the cardiovascular system. Meanwhile, it is highly appreciated that exosomes secreted by ESCs would recognize as alternative therapeutic strategies for treatment of CVDs. A study by Ratajczak et al. demonstrated that ESC-derived exosomes contain several stem cell-specific pluripotent molecules of Oct4 and Sox2 that support self-renewal and expansion of adult stem cells (Ratajczak et al., 2006). Proteome analysis by high performance liquid chromatography (HPLC) identified 857 proteins presented in exosomes derived from human ESC-derived MSCs. Of them, 20S proteasome is a candidate exosome protein that could synergize with other constituents to ameliorate tissue damage (Lai et al., 2012).

Recent studies suggest that exosomes from ESCs have potential to repair injured heart via inducing cardiomyocyte proliferation and promoting neovascularization, as well as exerting anti-apoptotic and anti-fibrotic properties. Lai et al. identified exosomes as the active cardioprotective component in the conditioned medium (CM) of human ESC-derived MSCs, indicating through the paracrine mechanisms (Lai et al., 2010). Kervadec et al. have proven exosomes secreted by human ESC-derived cardiovascular progenitors (hESC-Pg) produced equivalent benefits on cardioprotective effects as compared to hESC-Pg alone administration in a mouse post-infarct heart failure (HF) model. They subsequently identified 927 upregulated genes in the heart after treatment by hESC-Pg and their exosomes, of which 78% were associated with cardiac function (Kervadec et al., 2016). In another study, it was shown that treatment by human ESC-derived MSCs exosomes resulted in a reduction in infarct size after myocardial ischemia/reperfusion (MI/R) injury in mouse. It resulted in increased levels of ATP and NADH, decreased oxidative stress, and further increased phosphorylated (p)-Akt, p-GSK-3β, and reduced p-c-JNK in MI/R hearts (Arslan et al., 2013).

Particularly, several miRNAs already identified in ESCs derived exosome, play important roles in CVDs and stem cell transdifferentiation (Yuan et al., 2009; Chen and Lim, 2013). MiRNAs are short non-coding RNA molecules that inhibit gene expression by binding to complementary sequences at the 3′-UTR of their target gene transcripts (Bartel, 2004; Zhang et al., 2007). Khan et al. showed intramyocardial delivery of exosomes derived from mouse ESCs restored cardiac function in an acute myocardial infarction (MI) mouse model. Furthermore, they identified exosomes enriched miR-290-295 cluster, particularly of miR-294 were able to enhance neovascularization, improve cardiomyocyte survival and reduce fibrosis post infarction (Khan et al., 2015). All 3p-strands of miRNAs contain a common sequence of AAAGUGC in miR-290-295 cluster, locating at mouse chromosome 7. Whilst the enrichment of miR-294 in ESCs was also reported previously to exert positive effects on proliferation and negative effects on differentiation (Wang Y. et al., 2013; Guo et al., 2015). Unfortunately, the miR-290-295 cluster is not existed at all in human based on database in miRBase (http://www.mirbase.org/). Although exosomes from both ESCs and ESC-derived cells have analogous cardioprotective effects, exosomes from the ESC-derived cardiac lineage cells theoretically exert more protective effects by transferring the endogenous specific molecules to salvage the injured neighboring cells by regulating inflammation, apoptosis, fibrosis, and angiogenesis. Accordingly, these results encourage development of a great understanding of mechanisms associated with ESC-exosome therapy for CVDs.

### Exosomes from induced pluripotent stem cells (iPSCs) and IPS-derived cells

Induced pluripotent stem cell (iPSC), discovered by Shinya Yamanaka in 2006 (Takahashi and Yamanaka, 2006), is a type of pluripotent stem cells generated directly from adult cells by transfection of a cocktail of four specific genes encoding transcription factors including Oct3/4, Sox2, Kfl4, and c-Myc (Takahashi and Yamanaka, 2006; Takahashi et al., 2007). The beneficial effects of iPSCs transplantation have been demonstrated on cardiac repair and regeneration in mouse infarcted heart by enhancing cell survival and proliferation (Singla et al., 2011; Wong et al., 2013). Importantly, the iPSCs have no ethical issue but superior capacity of cardiomyocytes differentiation. Interestingly, the release of exosomes has proven to regulate nearby or distant cell‘s activities via paracrine mechanism. Huge interests have been gained to determine whether iPSC-derived exosomes can provide equivalent or even more benefits than iPSCs alone administration for treatment of CVDs.

Previous studies have demonstrated therapeutic efficacy of secreted vesicles including exosomes from iPSCs by affecting neovascularization and cardiomyocyte survival in experimental animal models of CVDs (Jung et al., 2017). Additionally, EVs secreted by human induced pluripotent stem cell-derived cardiovascular progenitors (iPSC-Pg) are effective in the treatment of CHF, possibly through their specific most of the 16 highly abundant miRNAs, which were evolutionarily conserved miRNAs and associated with tissue-repair pathways (El Harane et al., 2018). In a recent study, Adamiak et al. compared the safety and efficacy of iPSC-derived exosomes and iPSCs administration for cardiac repair *in vivo*. Both treatments exhibited improved left ventricle (LV) function. Interestingly, iPSC-derived exosomes exhibited superior cardiac repair with regard to LV function, vascularization, and amelioration of apoptosis and hypertrophy than iPSCs transplantation (Adamiak et al., 2017). Exosome-mediated information transfer has potential of regulatory effects on target cells. Particularly, exosomal transfer of miRNAs is an important process to regulate microenvironment during tissue regeneration. Wang et al. identified exosomes harvested from murine cardiac fibroblast (CF)-derived iPSCs contained cardioprotective miRNAs which include Nanog regulated miR-21 and hypoxia inducible factor 1α (HIF-1α) regulated miR-210. These secreted exosomes protected cardiomyocytes against H_2_O_2_-induced oxidative stress *in vitro* and MI/R injury *in vivo* (Wang Y. et al., 2015). In addition, Other studies have also shown the similar observation that cardiomyocytes enriched miR-21 and miR-210 alleviated oxidative stress-induced cardiomyocytes apoptosis (Zhu and Fan, 2012; Xiao et al., 2016; Diao et al., 2017). In addition to iPSC-exosomes, the iPSC-derivatives secreted exosomes have also exerted protective effects for the injured hearts, such as iPSC-derived MSCs (iPSC-MSCs) and iPSC-derived cardiomyocytes (iPSC-CMs) (Jung et al., 2017). For instance, exosomes and their cargo underlie the mechanism of action of iPSC-CMs in salvaging the injured cardiomyocytes in the peri-infarct region against apoptosis, necrosis, inflammation, remodeling and fibrosis (Yang, 2018). Furthermore, Hu et al. demonstrated that iPSC-MSC-derived exosomes activated angiogenesis-related gene expression, as well as promote human umbilical vein endothelial cells (HUVECs) migration, proliferation and tube formation (Hu et al., 2015). Zhang et al. found that transplanting human iPS-MSC-derived exosomes to wound sites resulted in accelerated re-epithelialization, reduced scar widths, and the promotion of collagen maturity (Zhang et al., 2015). Overall, these findings suggest that iPS-derived exosomes have been investigated widely in the field of cardiac regenerative medicine. Theoretically, patient specific iPSC/iPSC-derived cells can eliminate immunosuppression in the recipient. Therefore, these exosomes might be more useful for further application. However, significant challenges still exist for their clinical translation of iPSC-exosomes therapy in the future.

### Exosomes from heart-derived stem cells

It had believed initially that the heart is a terminally differentiated organ without any regenerative capacity for decades. However, recent studies provided evidence that heart contains stem cell populations with proliferative and regenerative capacity for repairing injured cardiomyocytes (Beltrami et al., 2001, 2003). Cardiac stem cells (CSCs), one type of tissue-specific adult stem cells, enhanced recovery of impaired cardiac function in ischemic hearts (Messina et al., 2004; Kim et al., 2013). It has become more and more clear that the injected CSCs exert their beneficial effects via the release of vesicles, particularly exosomes (Vandergriff et al., 2015; Prathipati et al., 2017). More importantly, exosome-based therapy could avoid the problems associated with traditional cell-based therapy. It is known that exosomes are natural secreted vesicles to deliver specific molecules from one cell to others. To study the functional benefits of CSC-derived exosomes, Vandergriff et al. injected exosomes via the tail vein in a mouse model of doxorubicin induced dilated cardiomyopathy. They observed inhibition of cellular apoptosis and fibrosis and subsequently improvement of impaired cardiac function (Vandergriff et al., 2015).

Cardiac progenitor cells (CPCs) hold great cardiac regeneration potential to improve heart functions (Zakharova et al., 2010; Ellison et al., 2013; Aminzadeh et al., 2015; Le and Chong, 2016). Research studies indicate the potential of CPC-derived exosomes as cell-free therapeutic for cardiac repair (Mol et al., 2017). CPC derived from the adult hearts comprise <1% of cells in the heart firstly described by Beltrami et al. (2003). Based on surface marker expression, researchers have identified multiple types of CPCs including c-kit+, Scal-1+, Isl-1+, cardiosphere-derived cells (CDCs) and cardiospheres (CSPs) (Beltrami et al., 2003; Oh et al., 2003). Both of CDCs and CSPs express endoglin known as CD105 (Smith et al., 2007). Using different isolated methods, we can separate and culture the cells individually. Importantly, all cells have a similar function with the potential to differentiate into multiple cardiac cell types, such as cardiomyocytes, vascular smooth muscle cells and endothelial cells. Previous study has identified that CPCs treatment as potential therapy to improve cardiac repair and prevent further damage in cardiac diseases (Liu et al., 2015). Indeed, exosomes derived CPCs carrying specific contents have been successfully used to treat CVDs. In a study by Chen et al., they determined Sca1+ CPC-derived exosomes are critical for cardiac repair by protecting against H_2_O_2_-induced H9C2 cardiomyocytes injury, which lead to approximately 53% reduction in cell apoptosis via inhibiting caspase-3/7 activation in a mouse model of acute MI/R (Chen et al., 2013). The pro-angiogenic property of CPC-derived exosomes was reported to stimulate migration of endothelial cells in a wound scratch assay (Vrijsen et al., 2010). Enrichment of miR-21 in Sca1+ CPC-derived exosomes act as significant communicators constantly shuttle between cells, which exerted beneficial effects on cardiac protection by targeting programmed cell death protein 4 (PDCD4) (Xiao et al., 2016). In addition, Gray et al. observed a diminished reparative capacity of Sca1+ CPC-derived exosomes when CPCs grown under normoxic conditions compared to exosomes from hypoxic cells. They further determined the mechanisms were as a result of an increase of proangiogenic miR-17 and miR-210 levels in exosomes under hypoxic conditions, resulting in enhanced tube formation of endothelial cells and decreased profibrotic genes expression in TGF-β stimulated fibroblasts (Gray et al., 2015).

To investigate whether the release of exosomes from CDCs contribute to cardiac repair, Ibrahim et al. injected CDC-secreted exosomes in a mouse injured heart after MI and found the beneficial effects of cardiomyocyte regeneration. Whereas, this effect was abrogated by inhibiting exosomes production with GW4869 (a neutral sphingomyelinase inhibitor) treatment. Thereafter, they observed the highly abundant miRNA-146a was partially contributor to this effect (Ibrahim et al., 2014). In pig models of acute MI, Gallet et al. determined open-chest intramyocardial (IM) delivery of human CDC-derived exosomes exert an effect on myocardial protection through increases of vessel density, and attenuation of adverse remodeling (Gallet et al., 2017). Additionally, Tseliou et al. described an indirect interaction between CSP-secreted exosomes and improved cardiac remodeling. They showed intramyocardial injection of CSP-derived exosomes-primed fibroblasts increased global pump function and vessel density, but reduced scar mass due to much higher levels of stromal-cell derived factor 1 (SDF-1) and vascular endothelial growth factor (VEGF) secreted by fibroblasts (Tseliou et al., 2015). These findings suggest the possible therapeutic use of exosomes from heart-derived stem cells as a novel strategy for treating CADs. Hence, we assume that exosomes from heart-derived cells, as a novel and potential therapy, may offer a great promise to improve cardiac repair and prevent damage on cardiac diseases.

### Exosomes from mesenchymal stem cells (MSCs)

Mesenchymal stem cell (MSC) is one type of multipotent stromal cells that can differentiate into a variety of cell types, which are emerging as an extremely promising and major cell-based therapeutic agent for tissue regeneration and repair (Haider and Ashraf, 2005; Wei et al., 2013; Gnecchi, 2016). However, Wilensky and their colleagues observed only 0.06% of the injected MSCs retained in the infarct zones of swine heart 14 days after acute MI (Freyman et al., 2006). In fact, *ex vivo* experimentation showed that both treatments by MSCs or MSC-CM significantly reduced MI/R injury, indicating at least partially through the paracrine mechanisms (Angoulvant et al., 2011). As an important component of paracrine systems secreted by MSCs, exosomes have been investigated in the field of cardiac regenerative medicine (Huang et al., 2015; Rani et al., 2015), indicating their potentials in treating CVDs. Interestingly, similar miRNA expression patterns have been shown in between MSCs and MSC-derived exosomes. While in an acute MI rat model, the comparable improved cardiac repair has been observed under both treatments, evidenced by increased cardiomyocyte proliferation, reduced apoptosis and inhibited fibrosis (Shao et al., 2017).

Among the various MSCs, bone marrow-derived MSCs (BMSCs) have been the most widely investigated in various diseases (Hoch and Leach, 2015). Beneficial effects of their exosomes administration have been demonstrated in different experimental animal models relevant to human cardiac diseases. Bian et al. collected EVs with around 100 nm in diameter secreted by BMSCs upon hypoxia stimulation. Thereafter, intramyocardial injection of these vesicles promoted angiogenesis and protected cardiac tissue from ischemic injury at least by enhanced blood vessel formation in a rat MI model (Bian et al., 2014). Furthermore, Teng et al. showed BMSC-derived exosomes significantly enhanced the tube formation, reduced infarct size and preserved cardiac systolic/diastolic performance after MI in rat (Teng et al., 2015). MiRNAs-containing exosomes secreted by BMSCs play important roles in regulating microenvironment in target cells during the process of cardiac regeneration. Feng et al. determined that miR-22 is highly enriched in exosomes secreted by mouse BMSCs after ischemic preconditioning, and administration of these exosomes significantly reduced infarct size and cardiac fibrosis by targeting methyl-CpG-binding protein 2 (Mecp2) in a mouse post-MI model (Feng et al., 2014). Other studies have also indicated miR-22 regulates endothelial inflammation, tissue injury, and angiogenesis (Hong et al., 2016; Gu et al., 2017). It has been known that transcriptional activator GATA-4 was involved in embryogenesis and myocardial differentiation, which has been proven to promote BMSC survival and differentiation in ischemic environments (Li et al., 2010; Yu et al., 2013). In a study by Yu et al., they determined the effects of exosomes derived from GATA-4 overexpressed BMSCs in a mouse MI model. The results showed these exosomes significantly increased cell survival and preserved mitochondrial membrane potential (MMP) in cardiomyocytes cultured under a hypoxic environment *in vitro* and restored cardiac contractile function and reduced infarct size in a mouse MI model. Subsequently they identified miR-19a was higher in GATA-4 overexpressed MSC-derived exosomes to involve in the cardioprotective effects by inhibiting phosphatase and tens in homolog (PTEN) (Yu et al., 2015). BMSC-exosomes have also shown to be indirectly associated with cardiac protective effects. It has been shown that miR-21-5p levels were significantly elevated in human engineered cardiac tissue (hECTs) treated with the exosome-enriched fraction of the hMSC-exo vs. untreated controls. In addition, knockdown of miR-21-5p in hMSCs significantly diminished exosomal enriched pro-contractile and associated calcium handling gene expression (e.g., SERCA2a and L-type calcium channel) effects on hECTs (Mayourian et al., 2018). In another study, Zhang et al. pretreated CSCs with 400 μg/mL MSC-derived exosomes for 24 h, and then injected CSCs to the peri-infarct zone of heart. The results revealed that in comparison with control CSCs, MSC-derived exosomes pretreatment significantly increased cell survival, enhanced capillary density, reduced cardiac fibrosis, and restored long-term cardiac function after MI in rat model (Zhang et al., 2016).

In a rat MI/R model, injection of BMSC-derived exosomes reduced apoptosis and myocardial infarct size and subsequently improved heart functions by inducing cardiomyocyte autophagy via AMPK/mTOR and Akt/mTOR pathways (Liu et al., 2017). In cecal ligation and puncture (CLP)-induced sepsis model, miR-223 highly containing BMSCs secreted exosomes confer protection against CLP-triggered cardiac dysfunction, apoptosis and inflammatory response via targeting SEMA-3A and STAT-3 (Wang X. et al., 2015). Besides that, the other favorable effects of BMSC-generated exosomes have also displayed on neurological recovery following stroke induction (Xin et al., 2013a; Doeppner et al., 2015). Xin et al. showed that intravenous administration of BMSC-exosomes improved functional recovery after stroke and benefited neurite remodeling, neurogenesis, and angiogenesis via transferring the contents with enrichment of miR-133b to astrocytes and neurons (Xin et al., 2013b).

In addition to BMSC-derived exosomes, other types of MSCs derived exosomes were also demonstrated beneficial effects on cardioprotection. Cui et al. demonstrated adipose-derived MSC (AdMSC)-derived exosomes led to a markedly increase in cell viability of H9C2 cells under hypoxia/reoxygenation (H/R) *in vitro*, and administration of AdMSC-derived exosomes protected ischemic myocardium from MI/R injury via activation of Wnt/β-catenin signaling *in vivo* (Cui et al., 2017). Furthermore, Wang et al. showed superior cardioprotective effects of endometrium-derived MSCs (EnMSCs) in a rat MI model as compared to BMSCs and AdMSCs. These differences may be caused by certain miRNAs particularly miR-21 enrichment in exosomes secreted from EnMSCs, which exerted effects on cell survival and angiogenesis by targeting PTEN (Wang K. et al., 2017). As noted above, these studies revealed critical roles of exosomes derived from MSCs in cardiovascular regeneration, which might be potential therapeutic interventions for treatments of CVDs. However, different MSC cell types secrete exosomes that carry specific sets of content exert diverse functional effects.

## Potential application of exosomes

Exosomes are critical cellular communicators between cells, which offer the clinical potential for both detection and treatment of various diseases. Noteworthy, exosomes are of great increasing interest for three major reasons with potential clinical applications in CVDs as following: (1) diagnostics and prognostic biomarkers, (2) therapeutic strategies, and (3) drug carriers (Dykes, 2017; Zhang et al., 2017).

### Exosomes as biomarkers of CVDs

Due to the relative easy and quick collection and measurement, secreted exosomes and their contents have successfully gained huge interests for their diagnostic and prognostic potentials in various diseases (Properzi et al., 2013; Yamashita et al., 2013; Khalyfa and Gozal, 2014; Xu et al., 2017). With respect to CVDs, transportation of cardiac and muscle-specific molecules in exosomes can also be rapidly detected in plasma holding great potential as specific prognostic biomarkers (Deddens et al., 2016). Moreover, exosomes enriched miRNAs hold great potential for predicting the risk of CVDs development. Li et al. identified plasma exosomal miR-422a and miR-125b-2-3p may serve as blood-based biomarkers for monitoring and diagnosing ischemic stroke (IS) patients (Li D. B. et al., 2017). Matsumoto et al. suggested that abundant p53-responsive miRNAs in exosomes from serum, such as miR-192, miR-194, and miR-34a were positively associated with increased likehood of acute MI patients for developing severe HF (Matsumoto et al., 2013). In another study, they enrolled 29 patients with acute coronary syndromes (ACS) and 42 with non-ASC. The results indicated that elevated serum levels of circulating cardiac-specific miR-1 and miR-133a in patients with ACS compared to non-ASC group. Notably, miR-133a was further identified in exosomes, and subsequently released into circulation (Kuwabara et al., 2011). Additionally, circulating exosomes enriched miR-146a levels are elevated in the plasma and hearts in peripartum cardiomyopathy (PPCM) patients (Halkein et al., 2013). Accordingly, the exosome-based biomarkers could provide important diagnostic and prognostic values for better management of patients. To improve our better understanding the potentials of exosomes as biomarkers in CVDs it is urgently required to investigate their diagnostic and prognostic values for CVDs in more prospective, multi-center, and large clinical trials.

### Clinical applications on exosome-based therapy

In addition to biomarkers, discoveries have identified that exosomes released from certain cell types exhibit therapeutic utilities in various diseases, including cancers (Inamdar et al., 2017), liver diseases (Masyuk et al., 2013), bronchopulmonary dysplasia (Matthay and Abman, 2017), and CVDs (Ibrahim et al., 2014; Chen G. H. et al., 2017; Zhang et al., 2017). It has been evident that exosomes and their contents have potentials to govern cell survival, proliferation, migration, and differentiation in the damaged heart (Loyer et al., 2014; Lewis et al., 2017). Chen et al. found exosomal lncRNA GAS5 regulated the apoptosis of macrophages and vascular endothelial cells, which might be an effective way for atherosclerosis therapy (Chen L. et al., 2017). Circulating elevated miR-146a enriched in exosomes resulted in a subsequent decrease in metabolic activity and decreased expression of Erbb4, Notch1, and Irak1 in PPCM patients (Halkein et al., 2013). Additionally, modified exosomes by Arg-Gly-Asp (RGD) peptide simultaneously possessed synergistic therapeutic angiogenesis effects, representing a potential for angiogenesis therapy (Wang J. et al., 2017). More importantly, as described in this review, stem cell-derived exosomes and their contents secreted under various pathological conditions, and this resultant therapies may exhibit superior beneficial effects and more safer than stem cell transplantation-based therapy in animal models such as MI, MI/R injury, HPH, and brain injury (Khan and Kishore, 2017). In summary, stem cell derived exosome-based therapeutics hold great promise for the development of therapy aimed at regenerating the damaged heart. Anyway, the exosomes deserve deeply to explore their functional roles and therapeutic efficiency, which prompt stem cell-derived exosome-based therapy would become an alternative promising strategy in replacement of stem cell therapy for clinical application of patients with CVDs.

### Potential application of exosomes in drug delivery

The unstable drugs *in vivo* environment poses significant challenges to successful therapeutic outcomes. Thus, drug delivery system has been importantly developed to optimize. Recent evidence showed exosomes, unlike other vectors for gene delivery, biologically derived nano-dimensional vesicle could be used to carry different contents to adjacent or distant targeted cells (Maheshwari et al., 2017). exosomes are emerging as a promising drug carrier selectively carrying factors especially several miRNAs in abundance and mediate crosstalk among different cell types, which provided much more potentials for therapeutic approach (Mittelbrunn et al., 2011; Mathiyalagan and Sahoo, 2017). Additionally, exosomes can avoid phagocytosis or degradation within the body. Interestingly, exosome-based delivery technique is thought to be an attractive tool to provide reliable evidence to overcome the inefficient and nonspecific delivery. Kim et al. have proven cancer-derived exosomes functioned as natural carriers that efficiently deliver their contents to cancer cells/tissues with the plasmids of CRISPR/Cas9, a therapeutic genome editing technology in a broad range of diseases (Kim et al., 2017). Similar with exosomes as drug carrier, it is notable that human Argonaute 2 (Ago 2) protein complexes are able to carry circulating miRNAs and protect them from degradation in human plasma, which may be a key effector protein of miRNA-mediated silence (Arroyo et al., 2011).

**Figure 2:**
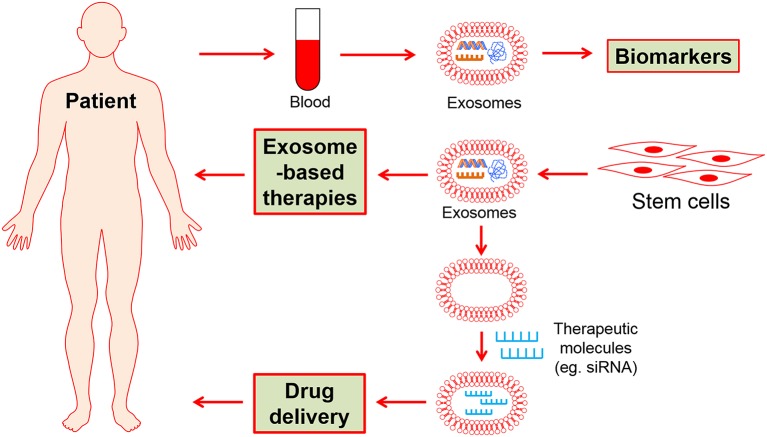
The potential clinical applications of stem cell-derived exosomes.

To date, the potential of stem cell exosome-based biomarkers, therapy strategies and drug delivery have largely explored specifically in the field of CVDs (Figure 2). It thus would fundamentally change the current therapeutic methods in the future.

## Conclusion and future perspectives

**Figure 3:**
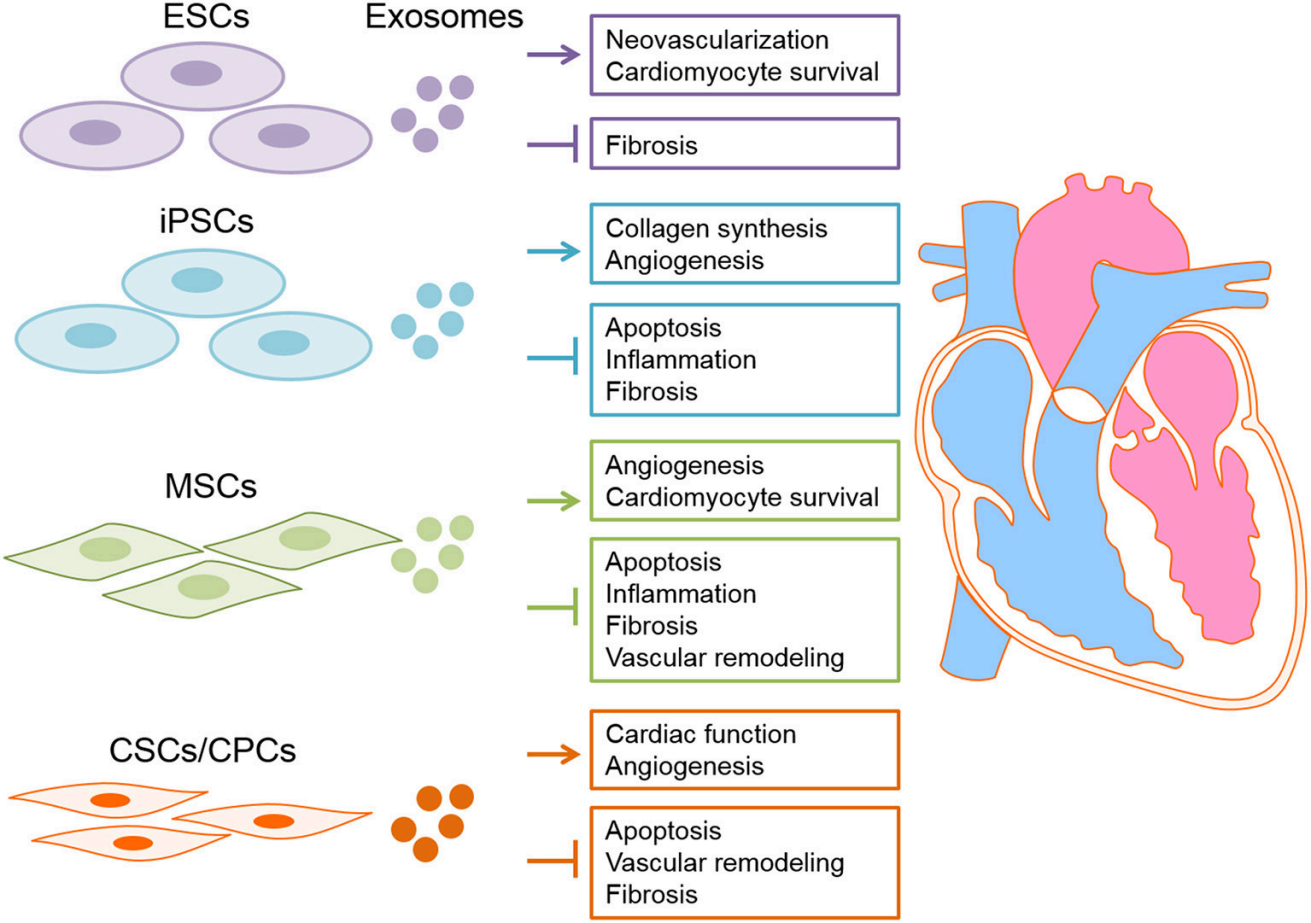
Exosomes derived from stem cells have proposed for cardiac disease therapy. Exosome derived from different types of stem cells, including embryonic stem cells (ESC), induced pluripotent stem cells (iPSCs), heart-derived stem cells and mesenchymal stem cells (MSCs) deliver nucleic acids (DNAs/RNAs) and proteins to the damaged heart tissue consequently exert cardioprotective effects.

In conclusion, exosomes derived from stem cells exerts beneficial effects on myocardial regeneration and thus possess a huge therapeutic capacity in prevention/treatment of CVDs (Table 2 and Figure 3). Accordingly, novel therapeutic strategies will provide translational perspectives for the clinical treatment of patients with CVDs in the future.

**Table 2:** Exosomes derived from different stem cell types and their roles in cardiac regeneration.

**Sources of exosomes**	**Animal model**	**Content**	**Exosome function**	**References**
**EXOSOMES FROM EMBRYONIC STEM CELLS (ESCS) AND ESC-DERIVED CELLS**				
ESCs	AMI	miR-290-295 cluster, particularly miR-294	Possess cardiac regeneration ability and modulate both cardiomyocyte and CPC based repair programs in the heart.	Khan et al., 2015
Human ESC-derived MSCs	MI/R	Unclear	increased levels of ATP and NADH, decreased oxidative stress, and further increased phosphorylated (p)-Akt, p-GSK-3β, and reduced p-c-JNK.	Arslan et al., 2013
Human ESC-derived cardiovascular progenitors	Heart Failure	927 upregulated genes in the heart after treatment by hESC-Pg and their exosomes	Cardio protective effects.	Kervadec et al., 2016
Human ECS-derived MSCs	MI	20S proteasome	Ameliorate tissue damage.	Lai et al., 2012
**EXOSOMES FROM INDUCED PLURIPOTENT STEM CELLS (IPSCS) AND IPS-DERIVED CELLS**				
iPSCs	MI/R	miR-21 and miR-210	Protect cardiomyocytes against H2O2-induced oxidative stress *in vitro* and MI/R injury *in vivo*.	Wang X. et al., 2015
iPS-MSCs	cutaneous wound healing	Unclear	Accelerate re-epithelialization, reduced scar widths, and the promotion of collagen maturity.	Zhang et al., 2015
iPSC-MSCs	mouse hind-limb ischemia model	Unclear	Activate angiogenesis-related gene expression, as well as promote human umbilical vein endothelial cells (HUVECs) migration, proliferation and tube formation.	Hu et al., 2015
iPSC-derived cardiomyocytes	Heart Failure	Unclear	Salvage the injured cardiomyocytes in the peri-infarct region against apoptosis, necrosis, inflammation, remodeling and fibrosis.	Yang, 2018
Human iPSC-derived cardiovascular progenitors	Heart Failure	16 highly abundant miRNAs	Increase cardiomyocytes survival, proliferation, and endothelial cell migration *in vitro* and improve cardiac function *in vivo*.	El Harane et al., 2018
**EXOSOMES FROM HEART-DERIVED STEM CELLS**				
Sca1+ CPCs	acute MI/R	Unclear	Protect against H2O2-induced H9C2 cardiomyocytes injury *in vitro* and reduce cell apoptosis *in vivo*.	Chen et al., 2013
Sca1+ CPCs	oxidative stress	miR-21	Exert beneficial effects on cardiac protection by targeting programmed cell death protein 4 (PDCD4).	Xiao et al., 2016
Sca1+ CPCs	TGF-β stimulated fibroblasts	miR-17 and miR-210	an increase of proangiogenic miR-17 and miR-210 levels in exosomes under hypoxic conditions, resulting in enhanced tube formation of endothelial cells and decreased profibrotic genes expression in TGF-β stimulated fibroblasts.	Gray et al., 2015
CDCs	MI	miR-146a	Beneficial effects of cardiomyocyte regeneration.	Halkein et al., 2013
CDCs	acute and chronic porcine MI	Unclear	Effect on myocardial protection through increases of vessel density, and attenuation of adverse remodeling.	Gallet et al., 2017
CSPs		SDF-1 and VEGF	Increase global pump function and vessel density, but reduced scar mass.	Tseliou et al., 2015
BMSCs upon hypoxia stimulation	MI	Unclear	Promote angiogenesis and protect cardiac tissue from ischemic injury at least by enhanced blood vessel formation.	Bian et al., 2014
BMSCs	MI	Unclear	Enhance the tube formation, reduced infarct size and preserved cardiac systolic/diastolic performance.	Teng et al., 2015
BMSCs after ischemic preconditioning	MI	miR-22	Reduce infarct size and cardiac fibrosis by targeting methyl-CpG-binding protein 2 (Mecp2).	Feng et al., 2014
**EXOSOMES FROM MESENCHYMAL STEM CELLS (MSCs)**				
GATA-4 overexpressed BMSCs	MI	miR-19a	Increase cell survival and preserved mitochondrial membrane potential in cardiomyocytes cultured under a hypoxic environment *in vitro* and restored cardiac contractile function and reduced infarct size *in vivo*.	Yu et al., 2015
Human MSCs		miR-21-5p	Increases of cardiac tissue contractility.	Mayourian et al., 2018
MSCs	MI	Unclear	Injection of CSCs pretreated with MSC-derived exosomes increased cell survival, enhanced capillary density, reduced cardiac fibrosis, and restored long-term cardiac function.	Zhang et al., 2016
BMSCs	MI/R	Unclear	Reduced apoptosis and myocardial infarct size and subsequently improved heart functions by inducing cardiomyocyte autophagy via AMPK/mTOR and Akt/mTOR pathways.	Liu et al., 2017
BMSCs	cecal ligation and puncture (CLP)-induced sepsis model	miR-223	Protection against CLP-triggered cardiac dysfunction, apoptosis and inflammatory response via targeting SEMA-3A and STAT-3.	Wang X. et al., 2015
BMSCs	middle cerebral artery occlusion strock model	miR-133b	Benefit neurite remodeling, neurogenesis, and angiogenesis.	Xin et al., 2013b
Adipose-derived MSC (AdMSC)	MI/R	Unclear	A markedly increase in cell viability of H9C2 cells under hypoxia/reoxygenation *in vitro*, and administration of AdMSC-derived exosomes protected ischemic myocardium from MI/R injury via activation of Wnt/β-catenin signaling *in vivo*.	Cui et al., 2017
Endometrium-derived MSCs (EnMSCs)	MI	miR-21	Superior cardioprotective effects of EnMSCs in a rat MI model as compared to BMSCs and AdMSCs.	Wang J. et al., 2017

Despite the growing amount of data have reported the beneficial effects and therapeutic potentials the field of CVDs, the exosome-based therapy in clinical application are still challenging and limited by several issues. Firstly, the large amount of time required and poor purified and isolated techniques failed to produce high quantity of exosomes limit their efficiency. (Tang et al., 2017). This will limit the future clinical use. Secondly, Since the exosome contained large number of bioactive factors, they could cause undesirable/side effects for heart tissue. Thirdly, due to heterogeneous components in exosomes, it may show the potential risk of tumor formation and the effects of immunogenicity based on nature of donor cells. Finally, it is unclear therapeutic effects of exosomes production under the special intervention during disease because of the complex structure of them. Accordingly, the investigations of stem cell-derived exosomes are situating in the primary stage. The variable composition and functional activity of naturally secreted exosomes impedes the pharmaceutical utilization. As reviewed above, it has become clear that exosomes derived from different conditions may contain different functional factors (nucleic acid and proteins etc.). This definitely decide their properties. The function and use of stem cell-derived exosomes are still in infancy and the more precise function of exosomes remains largely unknown. Therefore, there is a critical need for exploiting the functional roles of exosomes and their precise components that are critical for therapeutic delivery in the follow-up studies.

## Author contributions

WD, WM, and LZ read and collect the articles involved. JL organized the tables. YY and BC choose the topic of the review and write the manuscript. BC and ZD revise and has a final check for the manuscript.

### Conflict of interest statement

The authors declare that the research was conducted in the absence of any commercial or financial relationships that could be construed as a potential conflict of interest.

## Abbreviations

ACS: acute coronary syndromes
AdMSC: adipose-derived MSC
Ago 2: Argonaute 2
BM: bone marrow
CDCs: cardiosphere-derived cells
CF: cardiac fibroblast
CircRNAs: circular RNAs
CM: conditioned medium
CPCs: cardiac progenitor cells
CSPs: Cardiospheres
CVDs: cardiovascular diseases
EnMSCs: endometrium-derived MSCs
ESCs: embryonic stem cells
EVs: extracellular vesicles
HESC-Pg: human ESC-derived cardiovascular progenitors
HF: heart failure
HPH: hypoxic pulmonary hypertension
HUVECs: human umbilical vein endothelial cells
IHD: ischemic heart disease
IPSCs: induced pluripotent stem cells
IS: ischemic stroke
LncRNA: long noncoding RNA
LV: left ventricle
MiRNA: microRNA
MI: myocardial infarction
MI/R: myocardial ischemia/reperfusion
MSCs: mesenchymal stem cells
MVBs: multivesicular bodies.
